# The Responses of the Black Fungus *Cryomyces Antarcticus* to High Doses of Accelerated Helium Ions Radiation within Martian Regolith Simulants and Their Relevance for Mars

**DOI:** 10.3390/life10080130

**Published:** 2020-07-31

**Authors:** Claudia Pacelli, Alessia Cassaro, Lorenzo Aureli, Ralf Moeller, Akira Fujimori, Silvano Onofri

**Affiliations:** 1 Italian Space Agency, Via del Politecnico snc, 00133 Rome, Italy; claudia.pacelli@asi.it; 2 Department of Ecological and Biological Sciences, University of Tuscia, Largo dell’Università snc, 01100 Viterbo, Italy; Lorenzo.aureli@unitus.it (L.A.); onofri@unitus.it (S.O.); 3 German Aerospace Center, Institute of Aerospace Medicine, Radiation Biology Department, Space Microbiology Research Group, DLR, Linder Höhe, D-51147 Köln, Germany; ralf.moeller@dlr.de or; 4 Department of Natural Science, University of Applied Sciences Bonn-Rhein-Sieg (BRSU), von-Liebig-Straße 20, D-53359 Rheinbach, Germany; 5 Molecular and Cellular Radiation Biology Group, Department of Basic Medical Sciences for Radiation Damages, NIRS/QST, Chiba 263-8555, Japan; fujimori.akira@qst.go.jp

**Keywords:** Galactic Cosmic Rays (GCRs), Mars environment, black fungi, survival, UV-vis spectroscopy, resistance, melanin

## Abstract

One of the primary current astrobiological goals is to understand the limits of microbial resistance to extraterrestrial conditions. Much attention is paid to ionizing radiation, since it can prevent the preservation and spread of life outside the Earth. The aim of this research was to study the impact of accelerated He ions (150 MeV/n, up to 1 kGy) as a component of the galactic cosmic rays on the black fungus *C. antarcticus* when mixed with Antarctic sandstones—the substratum of its natural habitat—and two Martian regolith simulants, which mimics two different evolutionary stages of Mars. The high dose of 1 kGy was used to assess the effect of dose accumulation in dormant cells within minerals, under long-term irradiation estimated on a geological time scale. The data obtained suggests that viable Earth-like microorganisms can be preserved in the dormant state in the near-surface scenario for approximately 322.000 and 110.000 Earth years within Martian regolith that mimic early and present Mars environmental conditions, respectively. In addition, the results of the study indicate the possibility of maintaining traces within regolith, as demonstrated by the identification of melanin pigments through UltraViolet-visible (UV-vis) spectrophotometric approach.

## 1. Introduction

Mars is the prime target for the search of life beyond Earth, primarily because of the environmental conditions that have occurred in the past. Mars has evidence for past liquid water on the surface and an atmosphere that contains the essential elements for life [1,2]; moreover, the cold and dry conditions on the planet provide the opportunity that evidence for putative life may be well preserved. However, the Martian surface is characterized by an ionizing radiation environment significantly greater than that of Earth and therefore represents the major limitations for microbial survival in dormant state on present-day Mars. The thin Martian atmosphere and the absence of an ozone shield offer practically no protection against solar UV. The primary effects of UV radiation are a concern only on the surface of Mars, since the penetration of UV photons is limited to only a few micrometers [3] in the Martian near-subsurface or up to meters under a layer of snow or water [4]. The ionizing radiation of Solar Energetic Protons (SEP) and Galactic Cosmic Rays (GCR) dominate in the top few meters [3,4]. It has been measured by a Radiation Assessment Detector (RAD) instrument that the GCR dose rate that reached the Martian surface vary between 180–225 μGy/day [5]. The GCR spectrum is composed of 85% protons, 14% alpha particles (He ions), and a small fraction of heavy ions (fully ionized atomic nuclei). Ionizing radiation is measured in Gy, which is the total amount of energy deposited by ionizing radiation in a unit mass of the target material; different qualities of radiation can have different biological effectiveness, depending on their Linear Energy Transfer (LET) [6]. With increasing LET, it increases the density of ionization leading to a condensation of radiation effects in cells [5,6,7]. Densely ionizing radiation, such as the heavy ions and helium nuclei, can cause clusters of nearby DNA strand breaks, and so are particularly detrimental to cellular survival. Ionizing radiation can damage cellular components through direct deposition of radiation energy into biomolecules and indirectly by generating reactive oxygen species (ROS) [7]. While a metabolically active microorganism can repair the damages, the current freezing and water conditions in the Martian near-subsurface imply that any putative form of life could be dormant, so that the total dose accumulated over these periods could be crucial in determining cell survival [8]. Consequently, ionizing radiation imposes an upper boundary on the amount of time that a microbial organism can remain dormant in the near-subsurface of Mars. While a lot of experiments have been performed under simulated Martian conditions, mainly to study the microbial response to Martian UV, diurnal temperature shifts, and surface pressure [9,10,11], there has been relatively few investigations on the impact on ionizing radiation for (potential) microbial life on Mars. In natural Earth environments, microorganisms have capabilities to adapt to extreme conditions, including high dose of radiation uncommon on Earth, expanding the limits of habitability of Earth-like life beyond Earth. For example, melanized fungi are able to colonize environments with high radiation levels. A previous study [12] has reported that some fungal species are able to survive in the area around the Chernobyl reactor, growing towards the radiation source. Black fungi are also able to colonize spacecrafts that are considered high radiation environments [13].

The black fungus *C. antarcticus*, which thrives in the extreme environment of ice-free land of McMurdo Dry Valleys in Antarctica [14], has an unbelievable ability to resist physical extremes beyond its natural environment. Based on this extraordinary resistance, it has been widely used as test-organisms for astrobiological studies in both spaceflight and ground-based simulations. On board the European Space Agency (ESA) exposure facility EXPOSE E [15,16,17], dried colonies of *C. antarcticus* were exposed during 18 months to the extreme parameters encountered in space, including space vacuum, temperature cycles, and different spectral ranges of solar UV radiation, and also a simulated Mars environment in space (solar UV radiation 200–400 nm MJ/m^2^, cosmic radiation between 220–238 mGy and temperature cycles between −21.7 to +42.9 °C). Recent studies have also demonstrated its ability to resist a different kind of densely and sparsely ionizing radiation [18,19,20,21,22]. Part of this resistance was attributed to the presence of melanin in the cell-walls [20], the protection of extra-cellular polymeric substances [23], and probably the production of compatible endocellular cryoprotectants such as glycerol [24] and trehalose [25]. Here, in the frame of the STARLIFE irradiation campaign which aims to characterize the response of selected microorganisms to space-relevant radiation [26], we have analyzed the survival of *C. antarcticus* after irradiation with accelerated He ions, which is a component of the galactic cosmic spectrum. The goal was to address the following question: does radiation of accelerated He ions affect the survival of fungal cells within Mars regolith simulants? To answer this question, dried colonies of the fungus were mixed with Antarctic sandstones (the substratum in which they naturally live) and with two Mars regolith simulants, namely Phyllosilicatic Mars Regolith Simulant (P-MRS; early evolutionary stages of Mars) and Sulfatic Mars Regolith Simulant (S-MRS; late evolutionary stages of Mars) [27], and exposed to increasing doses of He ions. The post-exposure viability was assessed by both cultivation and molecular methods in terms of growing ability, DNA and plasmatic membrane integrity, and metabolic activity recovery. Moreover, the stability of melanin pigments, as component of fungal cell-wall, after radiation was checked by UV-vis spectroscopy.

## 2. Materials and Methods

### 2.1. Samples Preparation and Exposure Conditions

The Antarctic cryptoendolithic black fungus *Cryomyces antarcticus* CCFEE 515 was isolated by R. Ocampo-Friedmann from sandstone collected by H. Vishniac at Linnaeus Terrace (McMurdo Antarctic Dry Valleys, Southern Victoria Land) during the expedition in 1980–1981.

The fungus was isolated in Malt Extract Agar Petri Dishes as reported in [16], and since then it has been stored in the Culture Collection of Fungi From Extreme Environment (CCFEE) of University of Tuscia (Viterbo, Italy) that is a section of the Italian National Antarctic Museum. Firstly, a total of 2000 Colony-Forming Units (CFU) was spread from a 15 °C culture on Malt Extract Agar (MEA) medium (malt extract, powdered 30 g/L; agar 15 g/L; Applichem, GmbH) in Petri dishes, incubated at 15 °C for 3 months.

After growth, the colonies were desiccated under laminar flow in a sterile cabinet for one night. Once dried, colonies were mixed to three distinct materials and standard PCR tubes (200 µL volume) were totally filled with the different mixtures and fungal colonies. The mineral mixtures were previously dried-sterilized at 140 °C for 4 h.

Three sets of samples were thus investigated: i) colonies mixed with grinded Antarctic sandstone that is the Original Substratum (OS) where the fungus naturally occurs; ii) colonies mixed with P-MRS that mimics the regolith of phyllosilicate deposits mainly occurred on early Mars; iii) colonies mixed with S-MRS that is an analogue of regolith mainly observed in the current Martian sulphate deposits. The mineralogical composition of the Martian analogues is reported in Table S1 (from [28]). Samples were exposed to increasing doses of accelerated He ions (He^2+^, 150 MeV/n, LET in water: 2.2 keV/μm) at the Heavy Ion Medical Accelerator in Chiba (HIMAC) facility at the National Institute of Radiological Sciences (NIRS) in Japan. The applied doses were 50, 250, and 1000 Gy and the dose rate was 4.4 Gy/min; all samples were irradiated in a homogenous beam area. Laboratory controls (0 Gy) were kept in the laboratory at room temperature. All tests were performed in triplicate.

### 2.2. Survival Assessment

#### 2.2.1. Cultivation Test

Cultivation test was performed to determine the survival of fungal colonies after exposure to increasing doses of accelerated helium ions. Colonies from each set of samples were removed from the respective analogue with a sterile tweezer and rehydrated at 15 °C adding 1 mL of physiological solution (NaCl 0.9%) for 72 h.

The colonies were diluted to a final concentration of 50,000 CFU/mL, and 0.1 mL (5000 CFUs) for each sample was spread on MEA Petri dishes in quintuplicate. Samples were incubated at 15 °C for 3 months and the grown colonies were counted. Non-irradiated samples were kept in laboratory under room temperature and used as controls. In addition, dried colonies exposed without minerals to increasing doses of accelerated He ions from previous experiment [21] were used as reference and they are referred as un-shielded colonies.

#### 2.2.2. Membrane Damage Assessment

Quantitative PCR (qPCR) of DNA from samples treated with Propidium MonoAzide (PMA) was performed to assess the membrane integrity in samples. An aliquot of each rehydrated sample was added with 5 µL of a PMA solution (200 µM) and incubated in the dark for 1 h with constant shaking. Samples were then placed in ice and exposed to a halogen lamp for 10 min. PMA can selectively penetrate cells with damaged membranes and cross-link to DNA under exposure to light, thereby preventing Polymerase Chain Reaction (PCR). DNA extraction and purification were performed on PMA treated and untreated aliquots from each sample. DNAs were quantified and normalized at the same concentration (2 ng/mL) using the Qubit dsDNA HS Assay Kit (Thermo Fisher Scientific, Massachusetts, USA) and qPCR was performed to quantify the number of fungal internal transcribed spacer (ITS) ribosomal DNA fragments (281 bp) in both PMA-treated and untreated samples, according to [19]. All tests were performed in triplicate.

#### 2.2.3. Metabolic Activity Assessment by MTT (3-(4,5-dimethylthiazol-2-yl)-2,5-diphenyltetrazolium bromide) Assay

MTT assay was performed as a colorimetric test to evaluate metabolic activity of fungal cells. A total of 100 µL of cell suspensions (3.5 × 10^5^ cells/mL) in Phosphate-Buffered Saline (PBS), containing 0.5 mg/mL MTT salt (Thermo Fisher Scientific) were put into 96-well microplates. After incubation in the dark at room temperature for 48 and 72 h, MTT solution was removed with a multi-channel pipette and 100 µL of DiMethyl SulfOxide was added. The absorbance was read at 595 nm, and the average absorbance of wells containing only MTT was subtracted from the others. Means and standard deviations were calculated, and results were normalized with the laboratory controls.

#### 2.2.4. Statistical Analyses

Statistical analyses were performed by one-way analysis of variance (ANOVA) and pair wise multiple comparison procedure (Tukey’s test, [29]), carried out using the statistical software SigmaStat 2.0 (Jandel).

### 2.3. DNA Integrity Assessment

#### 2.3.1. DNA Extraction, Single Gene PCRs, and Random Amplified Polymorphic DNA Analysis

DNA extraction was performed on dried colonies using the NucleoSpin^®^ Plant kit (Macherey-Nagel, Düren, Germany) following the protocol optimized for fungi [18]. Quantitation of extracted genomic DNA was performed using the Qubit system and all the samples were diluted to the same concentration (2 ng/mL). Three overlapping tracts in the Internal Transcribed Spacer (ITS) regions and the Large SubUnit-coding Sequences (LSU) of the nuclear ribosomal RNA (rRNA) gene complex were amplified. The used primers were ITS4a (ATTTGAGCTGTTGCCGCTTCA), ITS5 (GGAAGTAAAAGTCGTAACAAGG), LR5 (TCCTGAGGGAAACTTC) and LR7 (TACTACCACCAAGATCT). PCR reactions were carried out for each sample in a solution consisting of 12.5 μL of BioMixTM (BioLine Ltd., London), 1 µL of each primer solution (5 pmol/μL), and 0.2 ng of DNA template, nuclease-free water was added until to reach the final volume of 25 µL. MyCycler Thermal Cycler (Bio-Rad Laboratories GmbH, Munich, Germany) equipped with a heated lid was used and amplification conditions are as reported in [30]. Relative band intensities were measured and compared by using Image Lab Software Version 6.1 (Bio-Rad, Hercules, CA, USA).

The whole genome integrity was assessed by Random Amplified Polymorphic DNA (RAPD). PCR reactions were carried out for each sample in a final solution containing 12.5 µL of BioMixTM, 5 pmol of primer (GGA)_7_ and 0.2 ng of DNA sample, in a final volume of 25 µL. Amplifications were performed according to [30].

#### 2.3.2. DNA Integrity by Quantitative qPCR Assay

Quantitative PCR was carried out to quantify the number of amplified ITS rDNA region by using LR0R and LR5 primers. The qPCR reactions were performed in triplicate with a solution containing 7.5 µL of qPCR cocktail (iQ SYBER Green Supermix, Biorad, MI, Italy), 1 µL of each primer solution (5 pmol/µL), and 0.1 ng of DNA template in a final volume of 15 μL. The amplifications were carried out by Biorad CFX96 real time PCR detection system.

### 2.4. Fungal Melanin Extraction and Spectrophotometric Analysis

Melanin was extracted from dried colonies of *C. antarcticus* grown on different substrata, following the protocol optimized for black fungi [31]. After the extraction, purified pigments were dissolved in 500 µL of NaOH 1M and its UV-visible spectrum was measured in VWR-UV 1600 PC Spectrophotometer by using M.Wave professional 2.0 software (VWR, Radnor, Pennsylvania, USA). A standard graph for estimation was used and was made using synthetic melanin. NaOH 1M was used as a blank and the instrument was set in a range of 200–800 nm for the analysis. The correlation between absorbance and wavelength was defined. To determine the concentration of extracted melanin, synthetic DHN (1,8-DiHydroxyNaphthalene) melanin (Thermo Fisher Scientific) was prepared in 1M NaOH at concentrations of 500 mg/mL and a standard curve at 650 nm was obtained as reported in [32].

## 3. Results

### 3.1. Cultivation Test

**Figure 1:**
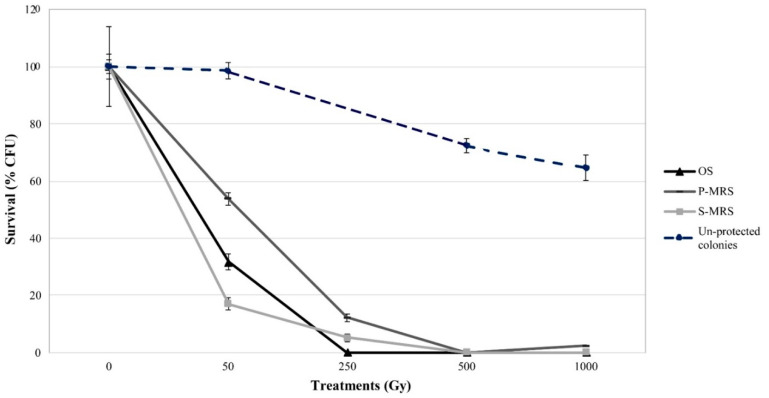
Cultivation test performed in colonies exposed to accelerated helium ions in presence of OS (Original Substrate) (black line), P-MRS (Phyllosilicatic Mars Regolith Simulant) analogue (grey line), and S-MRS (Sulfatic Mars Regolith Simulant) analogue (light grey line); compared with irradiated un-shielded colonies (dashed blue line; data from [21]).

The survivability of the black fungus *C. antarcticus* after irradiation with accelerated He ions was determined by counting the numbers of colonies formed on MEA plates, compared to the controls. Colonies survival was plotted as a function of the applied dose of accelerated He ions (Figure 1). Obtained data were compared with previous results, where the fungus was irradiated in absence of any substratum [21]. D_10_ values, the dose lethal for 90% of the initial colonies, were calculated from the survival curves, expressed by LogN values and are reported in Table 1.

Overall, survival results showed a common trend that is a fungal growth decrease as the radiation dose increases (Figure 1). Figure 1 (black line) shows the survival of *C. antarcticus* colonies mixed with OS substratum; appreciable growth ability up to 50 Gy, with a 31% of survivors, and loss of growth at the doses of 250 and 1000 Gy. Samples mixed with the P-MRS analogue showed a dose-dependent growth decrease, although the survival was reported even at the highest dose (6% of survivors at 1000 Gy; Figure 1, grey line). For colonies mixed in S-MRS analogue, the growth ability was maintained up to 250 Gy, with 6.5% of survivors (Figure 1, light grey line). No growth was recorded for samples irradiated with 1000 Gy.

### 3.2. Membrane Damage Assessment

**Figure 2:**
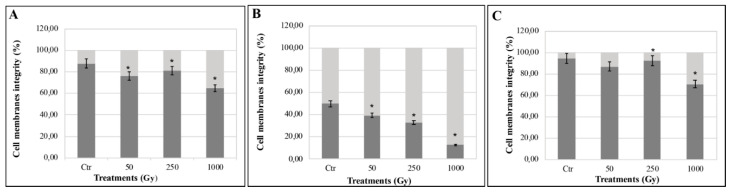
Percentage of intact and damaged cell-membranes measured with Propidium Mono Azide (PMA) assay coupled with qPCR of *C. antarcticus* mixed with (**A**) OS substratum, (**B**) P-MRS, and (**C**) S-MRS analogues. Light grey bars represent the percentage of cells with damaged cell membranes; dark grey bars represent the percentage of cells with un-damaged cell membranes. Significant differences were calculated by Tukey’s test with * *p* < 0.05.

The PMA assay coupled with qPCR was used to discriminate damaged cell-membranes to undamaged cell-membranes. PMA is a compound that can only penetrate cells with compromised cell membranes, binds DNA and prevents amplification. Figure 2A showed progressive cell-membranes damage with the increasing of irradiation for colonies mixed with OS substratum (up to 35.3% of damaged membranes at 1000 Gy). The doses of 50 Gy and 250 Gy did not show a significant difference compared to the control. *C. antarcticus* colonies mixed with P-MRS analogue (Figure 2B) maintained 12.87% of intact cell membranes at the highest dose (1000 Gy), while colonies mixed with S-MRS showed a low percentage of compromised membranes as 13.25 and 7.46% for 50 and 250 Gy, respectively (Figure 2C). However, non-statistically significant differences were found among 50 and 250 Gy irradiated samples, compared with the relative control. Around 30% of cells with damaged cell-membranes were recorded for colonies after irradiation at 1000 Gy.

### 3.3. Metabolic Activity Assessment by MTT Assay

**Figure 3:**
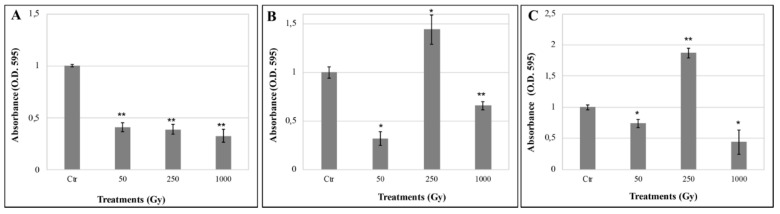
Effect of accelerated helium ions irradiation on metabolic activity recovery of *C. antarcticus* mixed with (**A**) OS substratum, (**B**) P-MRS, and (**C**) S-MRS analogues by MTT assay. Significant differences were calculated by Tukey’s test with * *p* < 0.05 and ** *p* < 0.001.

In metabolically active cells, MTT is reduced to a water-insoluble blue formazan by the succinate dehydrogenase, enzyme of the mitochondrial respiratory chain. The reduced product is quantified spectrophotometrically by dissolution in an organic solvent (DMSO), and the measured concentration is directly proportional to the number of metabolically active cells. Here, the MTT assay was performed to investigate to which extent cells are able to re-activate metabolic activity after the irradiation treatments. Figure 3 shows the metabolic activity recovery of irradiated cells after 48 h of rehydration. The MTT results of colonies mixed with OS substratum, demonstrated a decrease in *C. antarcticus* metabolic activity, with the increasing of He ions irradiation doses (Figure 3A). Formazan production of colonies mixed with P-MRS analogue (Figure 3B) revealed a high quantity at 250 Gy dose and a low formation at 50 and 1000 Gy doses, compared with the control. A similar trend for samples exposed at 250 Gy of accelerated helium ions has been found for samples mixed with S-MRS analogue (Figure 3C). However, a good amount of metabolic activity was reported at the highest tested dose of 1000 Gy in all samples, despite the analogues.

### 3.4. DNA Integrity Assessment

**Figure 4:**
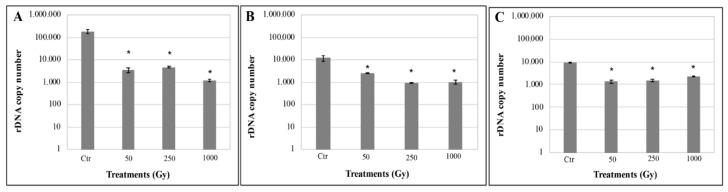
rDNA ((Internal Transcriber Spacer (ITS)-Large SubUnit region (LSU)) copy number quantification by qPCR of DNA of *C. antarcticus* colonies mixed with (**A**) OS substratum, (**B**) P-MRS, and (**C**) S-MRS analogues exposed to accelerated helium ions. Significant differences were calculated by Tukey’s test with * *p* < 0.05.

The amplified DNA fragments were obtained from amplification of ITS-LSU regions of 700, 1600, and 2000 bp, respectively. Figure 4A–C reported electrophoresis gels from amplification obtained from ITS5-ITS4a, ITS5-LR5, and ITS5-LR7 primers. Results showed no evident differences in DNA amplification among controls and irradiated samples of each sets. The whole genome was investigated by RAPD assay, based on PCR amplifications of genomic DNA, utilizing a short unspecific primer that is able to bind to different points in the genome and to reproduce a specific band pattern for the tested organism. The RAPD profiles were preserved in all the conditions tested (Figure S2). Any disappearance of high molecular weight band, that is expected in case of extensive DNA damage [33], was reported in the irradiated samples, indicating a good integrity of the whole DNA.

DNA damage analysis was performed by quantitative amplification of a gene, spanning the ribosomal LSU, of 939 bp length. Figure 4A–C showed the number of amplified DNA fragments after irradiation with increasing doses of accelerated helium ions mixed with the OS substratum, P-MRS, and S-MRS analogues, respectively. The results revealed a reduced DNA amplification while increasing the radiation doses for all tested samples. In overall, around 19,000 DNA copies were amplified on average and the amplification never goes less than 100 copies. Summarizing, all the gene amplifications worked out and the number of amplified DNA copies was never under the amplification limit.

### 3.5. Melanin Investigation by Spectrophotometric Analyses

**Figure 5:**
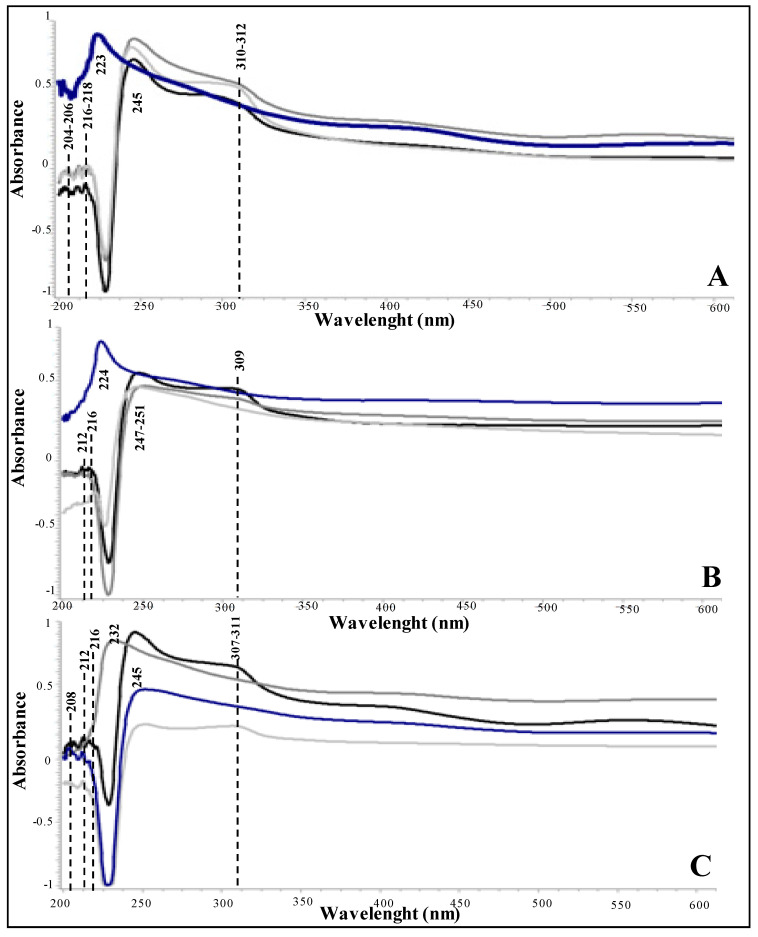
UV-Vis spectra of melanin pigment extracted from *C. antarcticus* colonies mixed with (**A**) OS substratum exposed to simulated space conditions and (**B**) P-MRS, and (**C**) S-MRS analogues and exposed to accelerated He ions at the doses of 50 Gy (black line), 250 Gy (grey line), 1000 Gy (light grey line), and the respective control (blue line).

Spectrophotometric analyses were performed on fungal melanin extracted from *C. antarcticus* colonies mixed with OS substratum and P-MRS and S-MRS analogues, after exposure to accelerated He ions. The wavelength of maximum absorbance was scanned at a range of 200 to 800 nm. The UV-visible absorbance spectrum of the purified pigments showed a strong absorbance in the UV region. The strong absorption in the UV region with a progressive decrease at high wavelengths is due to the presence of complex conjugated structures in the melanin molecule [34]. Figure 5 shows the melanin spectra obtained from colonies mixed with OS substratum (Figure 5A), P-MRS (Figure 5B) and S-MRS (Figure 5C) analogues, exposed to 50 (black lines), 250 (grey lines), and 1000 Gy (light grey lines), compared with the controls (blue lines). Changes in melanin absorbance has been reported in the irradiated samples, with an absorption peak around 245–251 nm; compared with relative controls, which shown an absorption peak at 223–224 nm, the typical wavelength of absorbance of *C. antarcticus* melanin pigments [31]. In addition, a bulge at ~310–312 nm was revealed only in exposed samples of OS set, in 50 Gy irradiated samples of P-MRS set, and 50 and 250 Gy irradiated samples of S-MRS sets, probably due to the presence of minerals during the UV-vis detection process. In fact, it could be representative of the goethite, which in general shows an UV absorbance near 330 nm, attributable to an Fe^3+^–O charge transfer [35,36].

In the initial part of the spectrum, which corresponds to lower wavelengths, the additional near 216–218 nm could be associated with the presence of montmorillonite, the main component (45%wt) of the P-MRS analogue [35,36], as further confirmation of the presence of mineral grains in the sample during the detection.

The concentration of extracted melanin was estimated by comparing the absorbance value at 650 nm of each melanin sample with the A_650_ standard curve of synthetic DHN melanin (Figure S3), and concentration values (mg/mL) were reported for all OS, P-MRS, and S-MRS samples in Table S2.

## 4. Discussion

The surface of Mars, unshielded by a thick atmosphere or by a global magnetic field, is exposed to high levels of cosmic radiation. This radiation environment is deleterious to the survival of dormant cells or spores and to the persistence of molecular biomarkers in the subsurface. The effects of ionizing radiation on Earth life is of high astrobiological interest. Among radiation, the direct effects of UV are a concern only on the surface of Mars, and the penetration of UV photons is limited to only a few micrometers [3,37]; while ionizing radiation has to be considered also for the subsurface. Indeed, Martian atmosphere is continuously traversed by GCR, which mainly consist of accelerated protons (about 87%) and helium ions (about 12%) [38]. These particles are the main radiation component both of the Martian radiative environments, either of the deep space. Primary GCR particles with energies that exceed the atmosphere cut-off, which has an average shielding depth of 23 g/cm^2^, hit the Martian surface. Here, due to the relative abundances of the particles and their different penetrating depths, accelerated protons and helium ions [39] dominate the radiation environment. Ions incident on the Martian surface can penetrate the top meters of regolith until attenuated [10]. Therefore, the shielding by the regolith against GCR may play a critical role in fostering the persistence of hypothetical forms of life in the subsurface environment of Mars. However, a decrease in the absorbed dose due to GCR particles is only observed from a few centimeters beneath the surface, without significant variations within the near-surface layer [40]. Therefore, this phenomenon makes GCR particles a critical hazard not only for any putative form of life colonizing the first few centimeters of Martian regolith, but also for the future space missions around the lunar orbit and towards Martian cruise.

In this context, the present experiment reproduced part of the radiation environment predicted in the loose dry regolith making up the shallowest layer of Martian surface, in order to assess its suitability to survival of terrestrial-like forms of life. To this purpose, we have exposed dry colonies of the cryptoendolithic black fungus *C. antarcticus* to increasing doses of accelerated He ions (up to 1 kGy), while mixed with Antarctic sandstones and two Martian regolith analogues. Indeed, accelerated He ions, together with protons, contribute to the major part of the absorbed dose induced by GCR in the surface and the shallow subsurface environments of Mars [40,41]. A study of the effect of elevated radiation doses, which significantly exceed the radiation on the surface of Mars (i.e., 76 mGy/yr) [5] as in the case of this experiment, is necessary for the extrapolation of biological effects on the geological time scale. Furthermore, in addition to ionizing radiation, the uppermost layer of the Martian subsurface is characterized by conditions of hyperaridity and extreme temperature variations. However, the presence of salts (namely perchlorates, sulphates, chlorides carbonates and nitrates [42], may let to a periodical formation of transient brines by deliquescence in the uppermost centimeters of the subsurface, thus making available limited amounts of liquid water in a latitude-dependent fashion [43]. Although no strong evidence of liquid water in an adequate amount to support terrestrial-like forms of life exists, the seasonality of the availability of shallow brines [44] would imply that any putative extant life in the near-surface environment is dormant (i.e., metabolically inactive) for long periods of time, thereby accumulating GCR doses over seasonal periods, until being reactivated by the presence of liquid water. Alternatively, the dormant state may expand over geological periods, until the cellular reactivation by global or local changes in the environmental parameters. Therefore, the total dose accumulated over these periods will be crucial in determining cell survival, and not the rate at which it is absorbed.

In our experiment, irradiation with up to 0.5 kGy of He ions, did not eradicate populations of *C. antarcticus* and did not induce high damage to either DNA or cell membranes.

The fungus survives doses up to 250, 1000, and 250 Gy when mixed with OS substratum and P-MRS and S-MRS analogues, respectively (Figure 1). These values are lower when compared with the same fungus exposed to the same He ions radiation without the minerals, where 70% of survivors were reported at the dose of 1000 Gy ([21], reported as reference in Figure 1, dashed line). This is more evident when looking at the D_10_ values (Table 1). The observation that fungi mixed with Antarctic sandstones and Martian regolith simulants were significantly more sensitive, with D_10_ values of 10, 667, and 227 Gy, respectively, than un-shielded fungal colonies (D_10_ = 5000 Gy, extrapolated data from [21]) to He ions radiation was quite surprising. This means that in the presence of Martian regolith simulants, instead of having protection, the number of secondary generated radiation, reduce the possibility for dormant fungal cells to persist in Martian subsurface environment of 10 times (Table 1). Given the shielding depths of the materials (i.e., OS = 1.48 g/cm^3^, P-MRS = 1.55 g/cm^3^, S-MRS = 1.53 g/cm^3^) and the parameters set for the accelerated ions (energy = 150 MeV/n, LET in water = 2.2 keV/μm), we can rule out that these results are due to the positioning of the cells within the Bragg peak region along the trajectory of the particles.

It can be suggested that fungal cells mixed with artificial Martian regolith, a complex mixture of various water-absorbing minerals (e.g., montmorillonite, kaolinite, hematite, anhydrite) were additionally affected by the generation of ROS such as peroxide, superoxide, or hydroxyl radicals in the Martian regolith simulants [45,46,47,48]. In our experiment, accelerated He ions radiation allows a re-activation of metabolic activity that was, however, not dose-dependent in *C. antarcticus*, as irradiation at 250 Gy led to an increase in metabolic activity in colonies mixed with P-MRS and S-MRS analogues (Figure 3). This would be explained by the fact that, depending on the extent of cellular damage, the ionizing radiation can either kill the cell or activate a response. In the latter case, one of the main cellular responses after radiation exposure is represented by mitochondrial biogenesis. The MTT assay for evaluating the metabolic activity is used to measure the mitochondrial activity, therefore an enhanced production of mitochondria as response to radiation exposure may lead to an increase of the general metabolic activity [49]. Accordingly, the increase of metabolic activity reported for cells mixed with P-MRS and S-MRS analogues (Figure 3B,C, respectively) can be attributed to an activated response of colonies to repair the radiation-caused damages after re-hydration.

Accelerated He ion radiation caused a certain amount of damage to cell membranes, with a decrease of membrane-intact cells by ~36%, 87%, and 30% at the dose of 1000 Gy in cells mixed with Antarctic sandstones, P-MRS, and S-MRS analogues, respectively (Figure 2).

DNA damage was not detectable at all *C. antarcticus* extracted DNA through single gene amplifications that were successful even at highest dose and longest gene sequences tested (Figure S1); accordingly, the fingerprinting profiles were perfectly maintained (Figure S2). We quantify the number of amplifiable DNA copies, and therefore, the presumably intact DNA molecules through qPCR; it resulted in few DNA degradation (likely caused by the formation of double strand breaks, DSBs) as shown by the reduced amplification efficiencies of DNA from irradiated cells in qPCR reactions (Figure 4). Nevertheless, an average of 1000 amplified DNA copies were reported even at 1 kGy exposure and even in samples where no survival was reported.

To put our results in the context of the possible survival of dormant Earth-like cells on Mars, we calculated the time necessary to inactivate 90% of fungal cells (see Supplementary material, Table S3). In the present experiment, we only considered the effects of accelerated He ions, which make up 12% of GCR spectrum [42]. The fluxes of He ions on the Martian surface were measured by the Radiation Assessment Detector (RAD) on the Mars Science Laboratory’s Curiosity rover over two distinct periods [39,42]; the GCR fluxes on Martian surface depends on the thickness of the atmosphere and the modulation by heliosphere, both of which vary periodically [47,50,51], and are not composed exclusively of α particles (similar to He ions), they also includes gamma rays and a large proportion of protons, as well as some heavier nuclei. However, a simplified model may be proposed which gives an approximation of the time that would be required by each fungal cell to be hit by as many particles (He ions) as used in the present experiment. He ions used in our experiment had energy of 150 MeV/nuc. Ions with energies lower than this value are more damaging, but less penetrating with very low fluxes. Therefore, in our estimation we considered only particles measured on Martian surface with energies above 135 MeV/nuc [42], and we calculated the estimated number of particles that hit the surface of each fungal cell depending on the exposure doses and the average size of fungal cells (around 35 µm). Despite the approximations of our simplified model, our results show as the time necessary to inactivate 90% of fungal cells in a hypothetical Martian surface and near-surface scenario may markedly vary depending on not only the depth in the regoliths at which cells are exposed, but also on the type of the mineral of the regoliths. We estimated that a dormant cell of *C. antarcticus* may be preserved in the surface of Mars for about 322.000 and 110.000 years within P-MRS and S-MRS analogues, respectively (see Table S2). Our calculations do not take into account the possibility that in some areas on Mars, temperatures above 0 °C and formation of liquid water are possible [45,52]. In such a case, active cells may be able to repair damages and to reproduce, then they can go in a dormant state until the next period of favorable environmental conditions (i.e., liquid water, warmer temperature) [53]. Considering this, we can assume an even longer preservation of putative Earth-like life on Mars.

The resistance of *C. antarcticus* to UV radiation doses uncommon for any habitat on Earth was suggested to be partially due to the presence of melanin pigments in the cell-wall [54], therefore we investigated the preservation of fungal melanin pigments through spectrophotometric approach. Results showed that irradiation with accelerated helium ions may alter the melanin structure. In Figure 5A–C is reported a shift of the typical melanin absorbance peak near 245–251 nm, with respect to *C. antarcticus* melanin pigment [31]. The additional peaks in the spectra (Figure 5A–C) revealed the presence of mineral grains during the detection. Overall, despite the presence of minerals, the detection of melanic pigments is always obtained. Due to their detection properties, it is possible to conclude that melanin pigments have high stability even in highly radiative environments and may have a double role in astrobiological research; their high radiation resistance has significant implications for the radioresistance of hypothetical life form on Mars and for the identification of potential biomarkers in the search for life beyond Earth. This greater radiation resistance has important implications for the estimation of potential survival times of microorganisms near the Martian surface and for the planetary protection issue.

## 5. Conclusions

In conclusion, the present study demonstrated the ability of the terrestrial black fungus *C. antarcticus*, to survive to accelerated He ions exposure in dehydrated form, when mixed with two Martian regolith simulants. Moreover, its biomolecules such as DNA and melanin are stable and detectable with our approaches. These results could have deep implications for future space missions, demonstrating the chance of long-term preservation of terrestrial-like microorganisms and their biomolecules on Mars.

## Figures and Tables

**Table 1:** D_10_ values = dose (Gy) of He ions irradiation leading to a 90% inactivation of the initial Coloning Forming Unit (CFU).

Samples	D_10_
Un-shielded colonies ^a^	5000
Colonies mixed with OS	101
Colonies mixed with P-MRS	667
Colonies mixed with S-MRS	227

^a^ Reference samples from [21]. OS = Original Substrate; P-MRS = Phyllosilicatic Mars Regolith Simulant; S-MRS = Sulfatic Mars Regolith Simulant.

## Supplementary Materials

The following are available online at https://www.mdpi.com/2075-1729/10/8/130/s1, Figure S1: PCR amplification of the A) ITS region (600 bp), B) ITS-LSU region (1600 bp), and C) ITS-LSU region (2000 bp) of samples of each set (OS substratum, P-MRS and S-MRS analogues). Pos Ctr: laboratory control; Ctr: Control; Neg Ctr: Negative Control; Figure S2: RAPD assay from *C. antarcticus* colonies extracted DNA, after exposure to accelerated helium ions irradiation. OS: Original substrate; P-MRS: Phyllosilicate Mars Regolith Simulant; and S-MRS: Sulfatic Mars Regolith Simulant; Figure S3: Positive correlation between synthetic DHN melanin at five concentrations and absorbance at 650 nm, used as standard curve for quantification according to [32]; Table S1: Concentration (in mg/mL) of extracted melanin from *C. antarcticus* colonies of exposed to accelerated helium ions. Table S2. Predictions of *C. antarcticus* survival period on the surface of Mars.
