# Supplementary material for: The Responses of the Black Fungus Cryomyces Antarcticus to High Doses of Accelerated Helium Ions Radiation within Martian Regolith Simulants and Their Relevance for Mars
Source: Life (Basel). 2020 Jul 31;10(8):130. doi: 10.3390/life10080130 (PMC7459982; doi:10.3390/life10080130)
Supplement: Supplementary file 1 [file life-10-00130-s001.pdf]

life

Supplementary Material for The Responses of The Black Fungus *Cryomyces Antarcticus* to High Doses of Accelerated Helium Ions Radiation within Martian Regolith Simulants and Their Relevance for Mars

Table S1. Phillosilicate Mars Regolith Simulant (P-MRS) and Sulfatic Mars Regolith Simulant (S-MRS) analogues composition (from [1]).

P-MRS		S-MRS	
**Mineral**	**Weight (%)**	**Mineral**	**Weight (%)**
Montmorillonite	45	Gabbro	32
Chamosite	20	Gypsum	30
Quartz	10	Dunite	15
Iron(III)-oxide	5	Hematite	13
Kaolinite	5	Goethite	7
Siderite	5	Quartz	3
Hydromagnesite	5		
Gabbro	3		
Dunite	2		

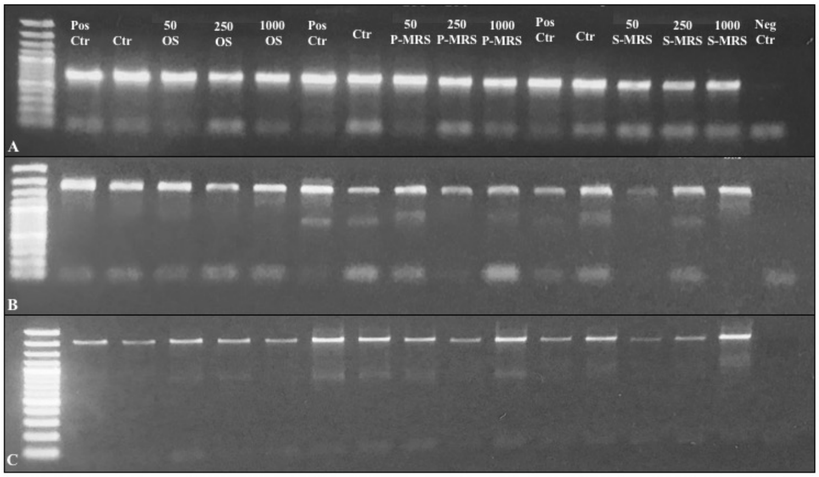

Figure S1. PCR amplification of the (**A**) Internal Transcriber Spacer (ITS) region (600 bp), (**B**) ITS- Large SubUnit region (LSU) region (1600 bp) and (**C**) ITS-LSU region (2000 bp) of samples of each set (Original Substrate (OS) substratum, Phillosilicate Mars Regolith Simulant (P-MRS) and Sulfatic Mars Regolith Simulant (S-MRS) analogues). Pos Ctr: laboratory control; Ctr: Control; Neg Ctr: Negative Control.

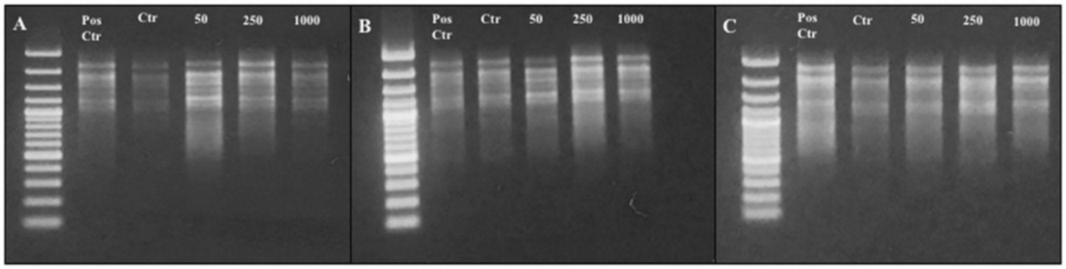

Figure S2. Random Amplification of Polymorphic DNA (RAPD) assay from *C. antarcticus* colonies extracted DNA, after exposure to accelerated helium ions irradiation. OS: Original substrate; P-MRS: Phyllosilicate Mars Regolith Simulant; and S-MRS: Sulfatic Mars Regolith Simulant.

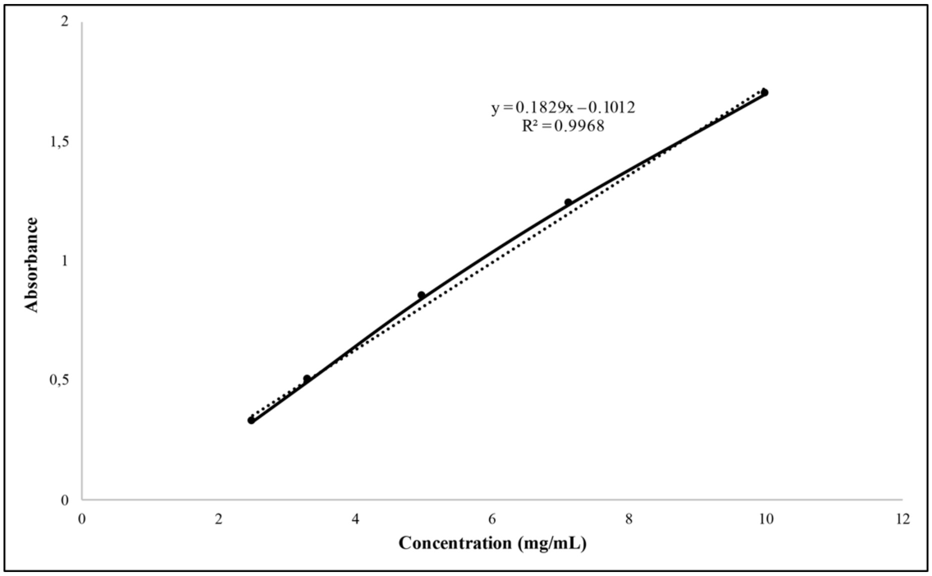

Figure S3. Correlation between synthetic 1,8-dihydroxynaphthalene (DHN)-melanin at five concentrations and absorbance at 650 nm used as standard curve for melanin quantification according to [2].

Table S2. Concentration (in mg/mL) of extracted melanin from *C. antarcticus* colonies of exposed to accelerated He ions.

Samples	Concentration (mg/mL)
OS Control	2.53
OS 50 Gy	1.08
OS 250 Gy	1.59
OS 1000 Gy	1.30
P-MRS Control	1.92
P-MRS 50 Gy	2.52
P-MRS 250 Gy	1.88
P-MRS 1000 Gy	5.47
S-MRS Control	1.35
S-MRS 50 Gy	1.16
S-MRS 250 Gy	5.11
S-MRS 1000 Gy	1.33

OS= Original Substrate; P-MRS= Phyllosilicatic Mars Regolith Simulant; S-MRS= Sulfatic Mars Regolith Simulant.

Calculation for Predictions of *C. antarcticus* Survival Period on the Surface of Mars

Based on survival results, we calculated the hypothetical survival time of cells of *C. antarcticus* on Mars considering the type of radiation and the doses applied in our experiment (Table S3). The second column shows the estimated number of particles that hit the surface of each fungal cells depending on the exposure doses in the experiment [3] and on the size of *C. antarcticus* cells (around 35 μm). The third column gives an approximation of the time required by each fungal cell to be hit by the corresponding number of particles on the Martian surface or near-surface environment. The values were calculated from the data reported by [4], measured in the presence of an average atmosphere thickness of 23 g/cm^2 and a solar modulation parameter of 550 MV. Only He isotopes (^{3}He and ^{4}He) with energies above 135 ± 15 MeV were considered. The fourth column shows the estimated Earth years required to inactivate 90% of fungal cells (D_{10}) within the three different substrata on Martian surface and shallow subsurface environment, considering only accelerated He nuclei as component of GCR.

Table S3. Predictions of *C. antarcticus* survival period on the surface of Mars.

Dose (Gray)	He Ions Fluence (Ions/Cell)	Time Exposure on Mars (Earth Years)	Estimated Survival* Time on Mars (Earth Years)
50 Gy	4970	24,236	OS = 48,835
250 Gy	24,815	121,010	P-MRS = 322,493
1000 Gy	99,260	484,041	S-MRS = 109,748

*Survival is intended as D_{10} values.

Reference:

1. Böttger, U.; De Vera, J.P.; Fritz, J.; Weber, I.; Hübers, H.W.; Schulze-Makuch, D. Optimizing the detection of carotene in cyanobacteria in a martian regolith analogue with a Raman spectrometer for the ExoMars mission. *Planet. Space Sci.* **2012**, *60*, 356–362, doi:10.1016/j.pss.2011.10.017.
2. Raman, N.M.; Ramasamy, S. Genetic validation and spectroscopic detailing of DHN-melanin extracted from an environmental fungus. *Biochem. Biophys. Rep.* **2017**, *12*, 98–107, doi:10.1016/j.bbrep.2017.08.008.
3. Alpen, E.L.; Powers-Risius, P.; Curtis, S.B.; DeGuzman, R.; Fry, R.J.M. Fluence-based relative biological effectiveness for charged particle carcinogenesis in mouse Harderian gland. *Adv. Space Res.* **1994**, *14*, 573–581.
4. Ehresmann, B.; Zeitlin, C.; Hassler, D.M.; Wimmer-Schweingruber, R.F.; Böhm, E.; Böttcher, S.; Brinza, D.E.; Burmeister, S.; Guo, J.; Köhler, J.; et al. Charged particle spectra obtained with the Mars Science Laboratory Radiation Assessment Detector (MSL/RAD) on the surface of Mars. *J. Geophys. Res. E Planets* **2014**, *119*, 468–479, doi:10.1002/2013JE004547.

© 2020 by the authors. Submitted for possible open access publication under the terms and conditions of the Creative Commons Attribution (CC BY) license (http://creativecommons.org/licenses/by/4.0/).
